# Environmental DNA Reveals Habitat Variables Driving Platypus (*Ornithorhynchus anatinus*) Distribution Across an Urbanised Landscape

**DOI:** 10.1002/ece3.70783

**Published:** 2025-01-09

**Authors:** Tamielle Brunt, Matt Cecil, Josh Griffiths, Christine Adams‐Hosking, Peter J. Murray, Annabel L. Smith

**Affiliations:** ^1^ School of Agriculture and Food Sustainability The University of Queensland Gatton Queensland Australia; ^2^ School of Environment The University of Queensland St Lucia Queensland Australia; ^3^ The Wildlife Preservation Society of Queensland Brisbane Queensland Australia; ^4^ EnviroDNA Pty Ltd Melbourne Victoria Australia; ^5^ The University of Southern Queensland Toowoomba Queensland Australia

**Keywords:** Anthropocene, aquatic biodiversity, climate change, freshwater ecology, multiple stressors, semiaquatic mammal

## Abstract

Freshwater waterways, and species that depend on them, are threatened by urbanisation and the consequences of the urban stream syndrome. In south‐east Queensland, Australia, little is known about the impacts of the urban stream syndrome on the platypus ( *Ornithorhynchus anatinus* ), meaning that populations cannot be adequately managed by conservation practitioners. The aim of this study was to determine how habitat and environmental variables, related to the urban stream syndrome, influenced platypus distribution across this region. We used environmental DNA sampling over a 5‐year period (2016–2020) to determine how platypus occurrence (presence or absence) was affected by habitat and the environment. Five scenarios based on key characteristics of the urban stream syndrome were modelled using binomial generalised linear models. The highest‐ranked model of platypus occurrence included positive effects of topographic wetness index and coarse organic matter. These findings reflect important waterway connectivity and food resources for platypus, highlighting relationships between platypus, their environment and their tolerance to urban stream threats. For example, platypuses are unlikely to occur in streams where water availability is reduced, and movement will be limited in shallow, dry streams. Therefore, waterway management should focus on water availability and connectivity, especially during drought. Our study can be used to guide management plans for the persistence of platypus and other freshwater species.

## Graphical abstract

The aim of this study was to determine how habitat and environmental variables related to the urban stream syndrome influenced platypus distribution across this region. The highest‐ranked model of platypus occurrence included positive effects of topographic wetness index and coarse organic matter. This relates to important waterway connectivity for platypus and highlights the relationships among platypus, their environment and their tolerance to urban stream threats. Our study can be used to guide management plans for the survival of the platypus and other freshwater species.

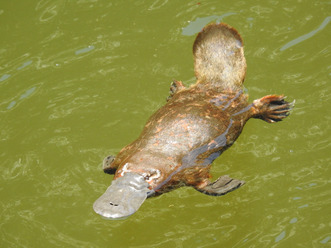

## 1 Introduction

Globally, freshwater ecosystems are threatened by urbanisation and climate change. Urbanisation causes land degradation, air and water pollution and biodiversity declines (Cresswell and Murphy 2017; Ferreira, Walsh, and Ferreira 2018; Olsson et al. 2019), while climate change influences the hydrologic cycle and water availability in freshwater ecosystems (Argent 2017; Konapala et al. 2020). In urban environments, when infrastructure encroaches on riparian zones, native vegetation is replaced with impervious surfaces. This results in a suite of consequences often called the ‘urban stream syndrome’. The urban stream syndrome is characterised by flashier water flow, increased nutrients and contaminants, altered channel morphology and stability, reduced biodiversity and an increase in the dominance of disturbance‐tolerant microbes, algae, invertebrate and macrophyte species (Paul and Meyer 2001; Meyer, Paul, and Taulbee 2005; Walsh et al. 2005). The collective impacts of these consequences degrade freshwater ecosystem function and the services which sustain biodiversity, such as water supply, filtration and regulation (de Groot et al. 2002; Vári et al. 2022). Understanding the complexity of these threats is critical to ensure they can be managed and freshwater ecosystems restored.

In addition to the urban stream syndrome, climate change is adding additional stress to freshwater ecosystems and freshwater‐dependent species. Precipitation patterns are predicted to become drier in some regions and wetter in others (Hale et al. 2016; Jia et al. 2019; Australian Bureau of Meteorology and CSIRO 2020), and increasing frequency and severity of drought threaten ecosystem stability (Reisinger et al. 2014; IPCC 2021). As air temperature increases, so do evaporation rates and changes in precipitation patterns, which affect water availability and disrupt flow regimes (Bunn and Arthington 2002; Bond, Lake, and Arthington 2008; Kernan 2015; Jiménez Cisneros et al. 2014; Jia et al. 2019). A lack of water and flow reduces connectivity leading to fragmentation of aquatic species' populations and potential loss of genetic diversity (Bond, Lake, and Arthington 2008; Cresswell and Murphy 2017). Air temperature also influences water temperature, which affects life history transitions such as the timing of breeding in fish, and physiology such as body temperature regulation in mammals (Robinson 1954; Cresswell, Janke, and Johnston 2021). Water temperature also influences aquatic macroinvertebrates and thereby food resources for many aquatic predators (Chessman 2012). The combined impacts of climate change and urbanisation in the context of the urban stream syndrome means that knowledge about contemporary habitats is required to predict extinction risk for freshwater‐dependent species.

The platypus, *Ornithorhynchus anatinus* , is known for its cryptic behaviour and reliance on freshwater ecosystems including water availability, earthen banks and macroinvertebrate prey (Grant 2007, 2014). It is a semiaquatic species that inhabits freshwater waterways along eastern Australia from Tasmania in the south to Cooktown in northern Queensland (Grant 1992; Carrick, Grant, and Temple‐Smith 2008). It is globally listed as near threatened on the International Union for Conservation of Nature Red List (Woinarski and Burbidge 2016). Key threats include modification of waterways by dams and weirs which alters flow regimes and reduces available surface water; and agriculture and urbanisation, which lead to habitat destruction, fragmentation and population isolation (Grant and Temple‐Smith 2003; Grant 2004, 2007; Lunney, Grant, and Matthews 2004; Griffiths et al. 2014; Hawke, Bino, and Kingsford 2019). Another threat is climate change, which affects the temperature and availability of their critical freshwater habitat (Klamt, Thompson, and Davis 2011). Platypus are phylogenetically unique, comprising one‐third of the world's monotreme biodiversity. It is also an apex predator in many Australian waterways, making its conservation substantially important (Winemiller, Humphries, and Pusey 2015; Hammerschlag et al. 2019).

**FIGURE 1:**
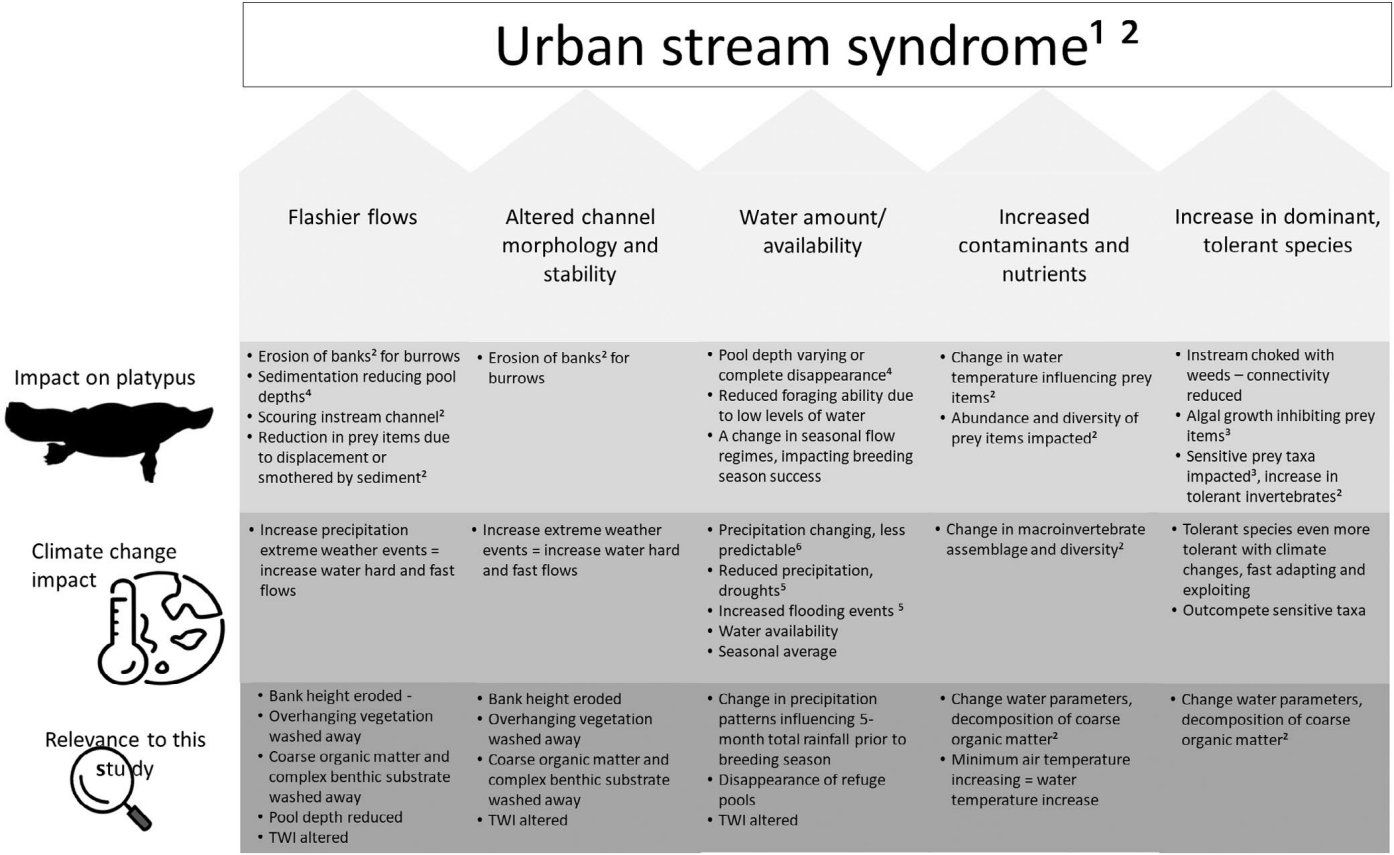
Conceptual model of urban stream syndrome impacts on platypus habitat resources, and effects of climate change. We expected negative effects on platypus occupancy to all of these changes in habitat and environment, based on knowledge of their habitat requirements. *Sources*: 1. Paul and Meyer (2001); 2. Walsh et al. (2005); 3. Serena and Pettigrove (2005); 4. Grant and Temple‐Smith (2003); 5. Reisinger et al. (2014); 6. Argent (2017).

Many regions in which platypus are found are rapidly urbanising, including south‐east Queensland, Australia. This region's estimated population of 3.1 million people is predicted to reach up to 4.9 million by 2041 (Queensland Government 2019, 2021; Australian Bureau of Statistics 2021). This will lead to new dwellings and infrastructure that will increase the urban stream syndrome stressors, with consequent threats to freshwater waterways and platypus populations (Figure 1). In the capital city of Brisbane, ephemeral urban streams have naturally low flow and flashy natural hydrology reflecting subtropical rainfall patterns of the region (McIntosh et al. 2013; Millington 2016). However, an increase in impervious structures in rapidly urbanising areas increases the velocity of water runoff, which increases erosion in the riparian zone and the stream channel where platypuses nest and forage (Walsh et al. 2005). This also produces sediment contaminants and deposition (Rohweder and Baverstock 1999; Grant and Temple‐Smith 2003; Serena and Pettigrove 2005; Martin et al. 2014). Sediment accumulates in pools, and sequentially affects benthic morphology (Grant and Temple‐Smith 2003; Walsh et al. 2005) and the ability of platypuses to forage efficiently (Serena and Pettigrove 2005).

In waterways, erosion and in‐stream scouring from stormwater channelling and sedimentation are driven by the removal of native vegetation and domestic stock grazing which results in hoof compaction of soil (Rohweder and Baverstock 1999; Grant and Temple‐Smith 2003; Boulton et al. 2014). These processes impact platypus burrowing sites by reducing bank stability, washing away or smothering prey and reducing pool depths with increased sediment (Grant and Temple‐Smith 2003; Martin et al. 2014). Weirs and dams have also modified waterways by changing water flows, creating barriers and altering species distributions and movements (Bunn and Arthington 2002; Kolomyjec et al. 2009; Furlan et al. 2013; Hawke, Bino, and Kingsford 2021). In some regions, this has caused genetic differentiation between platypus populations, which could affect genetic diversity and long‐term population viability (Mijangos et al. 2022). The extent to which urbanisation and climate change threaten platypus populations is unknown for this region and understanding the impact of these stressors will form the foundation of informed management decisions.

**FIGURE 2:**
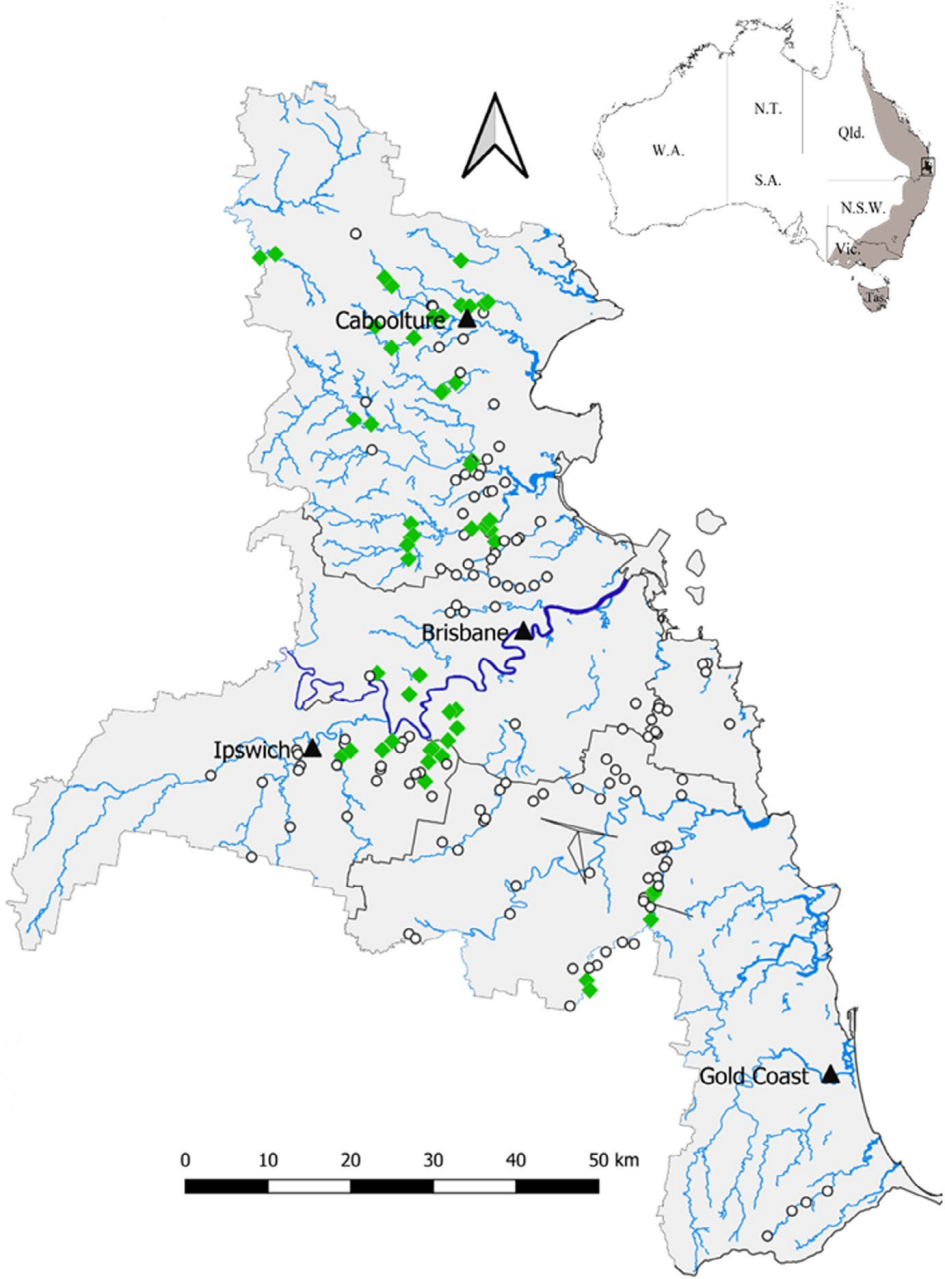
Water was sampled for platypus [ *Ornithorhynchus anatinus* ] eDNA at 185 sites across Greater Brisbane and the Gold Coast in south‐east Queensland, Australia. Platypus DNA was present at 42 sites (green diamonds), and absent at 143 sites (white circles). The inset shows platypus distribution in Australia (shaded area) and study region (boxed).

In this study, we explored possible drivers of platypus distribution across south‐east Queensland based on key conceptual aspects of the urban stream syndrome (Walsh et al. 2005) (Figure 1). Platypus occurrence data (presence or absence) were collected using environmental DNA (eDNA) across a region spanning 80 km^2^ (Figure 2). We used published literature on biologically relevant habitat and environmental variables to make predictions about how the urban stream syndrome stressors would affect platypus distribution (Figure 1). This formed the basis for our statistical models which tested effects of (1) flashier flows, (2) degradation of channel morphology and stability, (3) a decrease in water availability and overall flows, (4) an increase in contaminants and nutrients and (5) an increase in dominant, tolerant microbe, algae, invertebrate and macrophyte species. We predicted negative responses of platypus occupancy on these stressors. Our overarching aim was to develop new knowledge on key habitat and environmental drivers of platypus distribution to direct conservation resources appropriately. With increasing urbanisation and climate change, slowing habitat degradation and population declines are critical for the future survival of this iconic freshwater species.

## 2 Methods

### 2.1 Study Area

The study was conducted at 185 sites in 65 waterways across an 80 km^2^ area in the greater Brisbane and Gold Coast regions of south‐east Queensland, Australia (Figure 2). South‐east Queensland has a subtropical climate influenced by tropical systems from the north and fluctuations in the high‐pressure ridge to the south (Queensland Government 2020). Rainfall is highly variable, with an annual average of 1030 mm and variation driven by local factors, such as topography and vegetation, and broader‐scale weather patterns, such as the El Niño–southern oscillation (Queensland Government 2020). Most rainfall occurs in summer and autumn, with 388 mm and 295 mm on average per year, respectively. Seasonal average temperatures are 24°C in summer, 20°C in autumn/spring and 14°C in winter (Queensland Government 2020). Temperatures and the frequency of hotter days are predicted to increase for the region, while precipitation is predicted to decrease (Queensland Government 2020).

### 2.2 Site Selection

Sample locations were chosen using databases of previously documented platypus sightings since 1995 (Queensland Government 2017; Atlas of Living Australia 2017; Platypus Watch Network) and community‐based sightings of platypus (Figure 2). Within the vicinity of these locations, sample sites were selected based on physical access to the water body, water availability, water quality and water volume. We aimed for sites to be spatially independent by ensuring a minimum of 1 km between sample sites, based on the average home range size of a platypus (range = 0.2–7.3 km) (Grant 2007). Exceptions were made when a distinct barrier was present between sites, such as a weir or where pools were separated due to low flow. The mean distance between sites was 1.6 km (range 0.2–8.0 km). Some pairs of sites were separated by < 1 km, but these were all sampled within different years and thus assumed to be independent.

### 2.3 Water Sampling

Each year, from 2016 to 2020, water samples were collected for eDNA prior to and at the beginning of the platypus breeding season (June/July to October for Queensland, Grant 2007) when activity was expected to be the highest. The number of sites sampled per year ranged between 49 and 82 sites. Over the 5 years of sampling, some sites were repeatedly sampled across multiple years to increase the likelihood of getting a reliable detection estimate, especially when detections were negative in previous years (Table S1). However, only a single sample per site was included in the analysis (see below), giving a total of 185 sites across the study area. During a sampling session, two to three replicate water samples were taken at each site. Although high platypus detectability has been demonstrated from two samples (Lugg et al. 2018), we took three samples at some sites to increase detectability in larger waterbodies, such as rivers. Water samples of 110 to 500 mL (mean = 362 mL; standard deviation = 138 mL) were collected using a 60‐mL sterile syringe and were immediately filtered through a 0.22‐μm filter (Sterivex, Merck, Germany). Filters were stored and transported at 4°C and sent to the processing laboratory within 48 h to minimise the risk of eDNA degradation (Goldberg et al. 2016; Hinlo et al. 2017; Lance et al. 2017; Lamb et al. 2022). Care was taken to minimise eDNA contamination between sites by wearing latex gloves, using new sampling equipment at each site and avoiding the transfer of soil, water and organic material between sites.

### 2.4 DNA Extraction and Amplification

Molecular methods followed Lugg et al. (2018). In brief, DNA was extracted from the filters using a Qiagen DNeasy Blood and Tissue kit (QIAGEN, Chadstone, Australia). Species‐specific primers and a TaqMan minor groove binding (MGB) probe were developed to target a 57 bp fragment of the mitochondrial control region based on existing sequences of individuals from across the species range (GenBank accessions: HQ379855–HQ379928). The primers and probe were a custom TaqMan gene expression assay: forward primer OAcr_F CAGCAATACCCTAGACAAGG; reverse primer OAcr_R CGCTTCAATGGCTGCGC; and MGB probe OAcr_MGB CGAACCCCATGAGTAGAAAAT. Primer specificity was checked using a Blast search of the NCBI nucleotide database, with no close matches found outside of *O. anatinus* .

Real‐time TaqMan PCR assays were conducted using a Roche LightCycler 480 system in a 384‐well format. Ten μL reactions containing 5 μL of 2 × Qiagen multiplex PCR Master Mix (Qiagen), 0.5 μL 20 × TaqMan Gene Expression Assay, 2.5 μL ddH2O and 2 μL of DNA were prepared in triplicate. Control reactions were included in each 384‐well assay plate containing 10, 100, 1000, 10,000 and 100,000 copies of *O. anatinus* CytB synthetic oligonucleotide and a negative control with no DNA template. The qPCR efficiency determined from the *O. anatinus* CytB synthetic oligonucleotide standard curve was 100.3%. Amplification conditions were 15 min at 95°C, followed by 50 cycles of 15 s at 95°C and 1 min at 60°C. Amplification profiles of each PCR were used to determine the crossing point (Cp) value using the absolute quantification module of the LightCycler 480 software package. A TaqMan Exogenous Internal Positive Control VIC probe was run for each sample to test for the presence of PCR inhibitors. All extractions and qPCR analyses were undertaken in a room dedicated to low‐quantity DNA sources.

### 2.5 Habitat Quality Index and Environmental Variables

For each site, we compiled data on habitat (local and site level) and environmental (larger‐scale) variables which have been reported to affect platypus presence (Table S2). These variables were associated with platypus shelter, their ability to successfully mate and ability to forage efficiently.

Habitat variables at every site were measured during field surveys using a rapid visual assessment of an approximately 40‐m stream section based on a habitat quality index (Grant 2014) (Table S3). Seven riparian habitat variables and four in‐stream variables were chosen based on those identified from previous research to influence platypus presence (Table S2). For each variable, a score between 0 and 4 was recorded, which related to a percentage of the site (0 = 0%, 1 = 1%–25%, 2 = 26%–50%, 3 = 51%–75% and 4 = 76%–100%). The 11 habitat variables were grouped into broad categories, including bank (bank height, earthen banks, absence of erosion, consolidated banks and concave or near vertical banks), water (water depth), vegetation (native large‐medium sized trees on banks, overhanging vegetation, in‐stream large woody debris and coarse organic matter) and substrate (cobbled and complex benthic substrate) characteristics (Table S2). In this study, none of the habitat variables recorded a score of 0. As such, the potential for bias or inflated Type II errors due to a unique ‘0’ class does not apply to our data (Verhulst and Neale 2021). While the habitat quality index scoring system includes a provision for a ‘0’ category to account for the absence of a habitat feature, this was not observed at any of the surveyed sites. Therefore, the classification effectively ranged from 1 to 4, representing varying degrees of habitat feature presence.

Six environmental variables were collected from weather station databases, according to their potential to influence platypus distribution (Table S2). Precipitation data included total annual rainfall and antecedent rainfall (total 5‐month rainfall prior to breeding season) (Australian Bureau of Meteorology 2020a). Temperature data included minimum and maximum annual air temperature (Australian Bureau of Meteorology 2020b) collated for the study period (2016–2020) and extracted from gridded ASC files using the R package ‘rgdal’ (2021). Topographic wetness index (Gallant and Austin 2012) and elevation (Hutchinson et al. 2008) were extracted using Esri ArcGIS 10.8 (2019). The extracted values of TWI ranged from 5 to 23, with the larger value indicating higher wetness. Elevation ranged from 4 to 183 m above sea level.

### 2.6 Analyses

The majority of the 185 sites were sampled once (62%, *n* = 114) and the remaining sites were sampled more than once (38%, *n* = 71), two to five times during the 5‐year study (Table S1). Our final dataset comprised the first recorded detection from each site, so that each site was only included once, with sites considered temporally independent. This allowed us to model population‐level year effects across the entire study region.

Binomial generalised linear models (GLM) with a logit‐link function (R Core Team 2021) were used in a model selection framework to analyse the probability of platypus occurrence over 5 years, in response to 11 habitat and six environmental variables. All independent variables including the habitat quality index between 0 and 4 were scaled (x‐mean(x)/SD(x)). There were correlations among some variables, so we first identified a single uncorrelated variable from each variable group (precipitation, temperature, bank, water depth and vegetation, Table S2) for inclusion in the final set of models. Our approach was to develop a small series of models based on different aspects of the urban stream syndrome and their likely influence on platypus habitat (Figure 1) (Burnham and Anderson 2002). We did not conduct model averaging because it biases parameter estimates, particularly when interactions are present (Freckleton 2011; Hegyi and Garamszegi 2011) but we made inferences from all models with a ΔAICc ≤ 2 (Arnold 2010). We did not attempt to fit all combinations of the 17 variables or simplify our general models because it would have generated an impractical number of combinations which would be computationally challenging and biologically incomprehensible (Guthery 2008).

Prior to modelling, we conducted Pearson correlation tests between all pairs of 17 habitat and environmental variables and considered those with *r* > 0.30 to be correlated (Figure S1). This was a more stringent correlation threshold than is often used (e.g., *r* > 0.7, Dormann et al. 2013) because many variables were related to the same physical features or processes (e.g., earthen banks and consolidated banks, Table S2) and we wanted to avoid overinflating the importance of these variables. One variable from each group was selected to represent that variable group based on the one with the strongest potential influence on platypus distribution (Table 1). Of the correlated bank variables (bank height, concave/near vertical, consolidated, earth and absence of erosion), bank height was selected because platypus require a high bank to burrow (Grant 2007; Brunt, Adams‐Hosking, and Murray 2018). Of the rainfall variables, antecedent rainfall (rainfall total in the 5 months prior to sampling) was selected because it influences platypus prior to the breeding season (Serena et al. 2014). Overhanging vegetation was selected for its importance for bank stabilisation and protection, shelter, foraging and concealing burrow entrances (Serena et al. 1998; Grant 2014). Pool depth was included due to the positive association with platypus occurrence given their reliance on water (Grant 2007, 2014; Brunt, Adams‐Hosking, and Murray 2018) as well as the association with the urban stream syndrome. Minimum and maximum air temperatures were not correlated with each other (*r* < 0.30) or any other variable and were both included in the models (Figure S1). Nine uncorrelated habitat and environmental variables were used in subsequent analyses: coarse organic matter, bank height, pool depth, overhanging vegetation, complex benthic substrate, minimum and maximum air temperatures, antecedent rainfall and TWI.

**TABLE 1:** Candidate models for examining effects of the urban stream syndrome on platypus ( *Ornithorhynchus anatinus* ) in south‐east Queensland, Australia.

Urban stream syndrome model	COM	CBS	MinT	OV	BH	PD	TWI	AR	Year	Year^2^	CBS:year
Null	−	−	−	−	−	−	−	−	−	−	−
Channel morphology	+	+	−	+	+	−	+	−	+	+	+
Flashier flows	+	+	−	+	+	+	+	−	+	+	+
Dominant and tolerant species	+	−	+	−	−	−	−	−	+	+	−
Nutrients and contaminants	+	−	+	−	−	−	−	−	+	+	−
Water availability	−	−	+	−	−	+	+	+	+	+	−

*Note:* Variables included in each model are indicated by +. A null model was included as a baseline against which to examine the strength of the urban stream syndrome models.

Abbreviations: AR, antecedent rainfall; BH, bank height; CBS, complex benthic substrate; COM, coarse organic matter; MinT, minimum temperature; OV, overhanging vegetation; PD, pool depth; TWI, Topographic Wetness Index.

The nine chosen habitat variables were then fitted in five separate GLMs based on the urban stream syndrome: (1) channel morphology and stability, (2) flashier flows, (3) increase in dominant, tolerant species, (4) increased nutrients and contamination and (5) a decrease in water amount and availability (Figure 1 and Table 1). A null model was included in the candidate model set as a baseline against which to examine the strength of the urban stream syndrome models.

We used a binomial GLM to model population‐wide year effects across the entire study region. Preliminary data exploration indicated a nonlinear, unimodal response of platypus occurrence to sampling year; thus, year and its quadratic form (year^2^) were fitted in all models. To determine whether the response to each habitat and environmental variable changed over time, we conducted preliminary model selection to examine interactions with the year variable. For each of the five urban stream syndrome categories, we began with a model including all variables and no interactions. We then fitted year in an interaction with the habitat and environmental variables within this ‘base’ model, resulting in three to seven models per urban stream category. For each category, each pair of models (the interaction and non‐interaction model) was ranked using the second‐order Akaike information criterion (AICc) (Hurvich and Tsai 1989; Tredennick et al. 2021) and, if the interaction model improved the fit of the base model by ΔAICc > 2, it was selected for the final modelling stage.

The final five models were then ranked using AICc to determine which urban stream syndrome characteristics had the strongest effect on platypus distribution. We considered two models ranked within AICc < 2 to have similar support from the data (Hegyi and Garamszegi 2011). We calculated effect sizes and confidence intervals of all terms within each model to examine their importance. We considered a term important if the confidence interval around the mean did not overlap with zero.

## 3 Results

**FIGURE 3:**
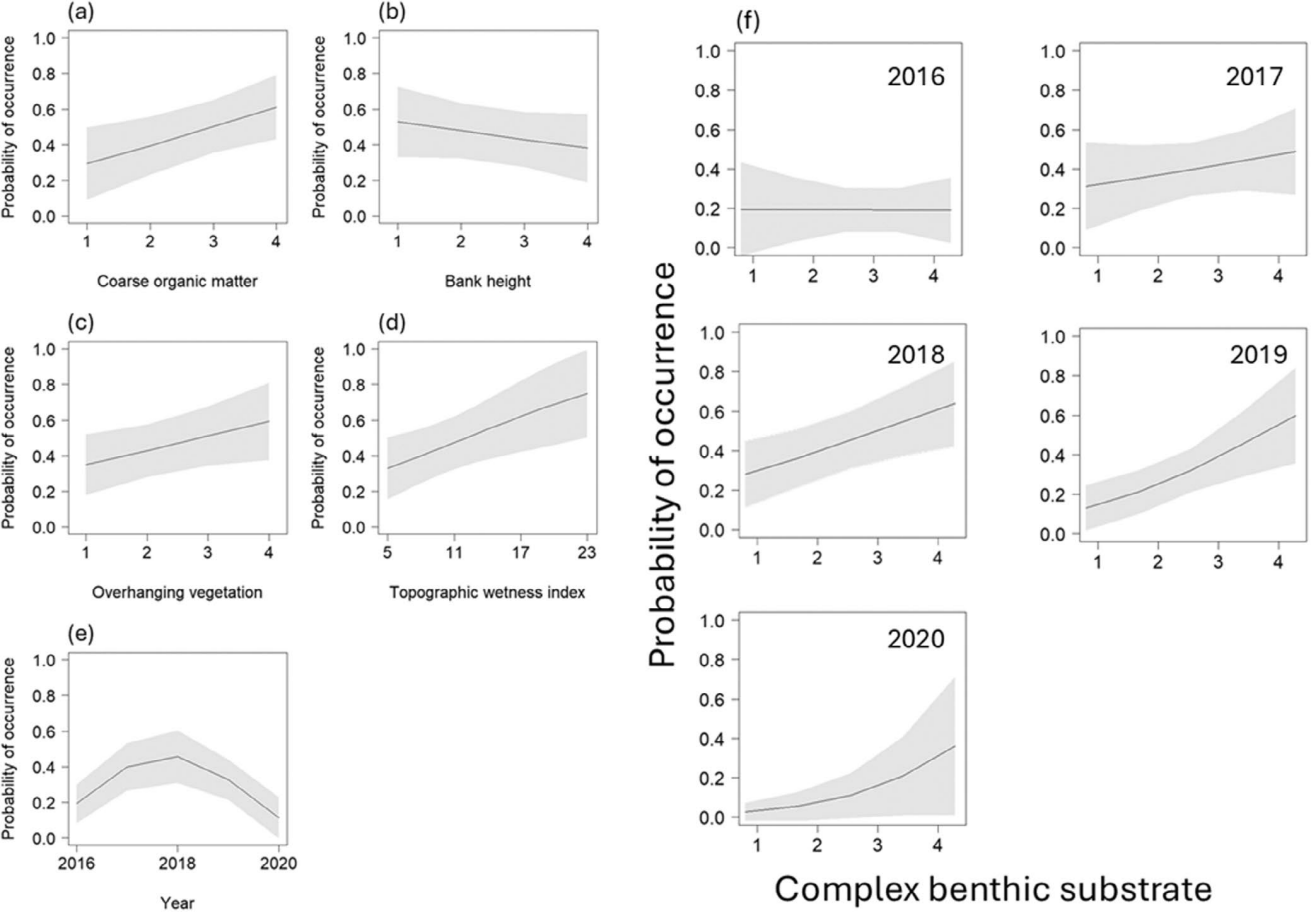
Model estimates and 95% confidence intervals from the first‐ranked model of the probability of platypus ( *Ornithorhynchus anatinus* ) occurrence, including variables representing channel morphology and stability. The model included effects of (a) coarse organic matter, (b) bank height, (c) overhanging vegetation, (d) topographic wetness index, (e) year and (f) an interaction between year and complex benthic substrate. The effect size for topographic wetness index indicated it was important, while the other variables had weaker effects (Figure 4a; Table S4).

Platypus were detected at 54 of the 185 sites (29%, Figure 2). All models were ranked higher than the null model (ΔAICc > 2, Table 2). The channel morphology model was the highest ranked and fit the data better than the other models (ΔAICc > 2, Table 2). The first‐ranked model held 58% of the weight and included variables related to channel morphology and stability: coarse organic matter, bank height, overhanging vegetation, TWI, year and complex benthic substrate (Figure 3, Table 2). None of the other models were ranked within Δ AICc < 2 of the first‐ranked model, although the flashier flows model which included pool depth was close (Δ AICc = 2.17, Figure S2).

**TABLE 2:** Model selection results examining urban stream syndrome effects on the probability of platypus ( *Ornithorhynchus anatinus* ) occurrence, ranked by the second order Akaike Information Criterion (AICc).

Model	Degrees of freedom	Log−likelihood	AICc	ΔAICc	AICc weight
Channel morphology	9	−94.708	208.4	0.00	0.579
Flashier flows	10	−94.677	210.6	2.17	0.195
Dominant and tolerant species	4	−101.774	211.8	3.33	0.110
Nutrients and contaminants	5	−100.722	211.8	3.34	0.109
Water availability	7	−101.324	217.3	8.84	0.007
Null	1	−111.710	225.4	17.00	0.000

*Note:* Terms included in each model are shown Table 1.

**FIGURE 4:**
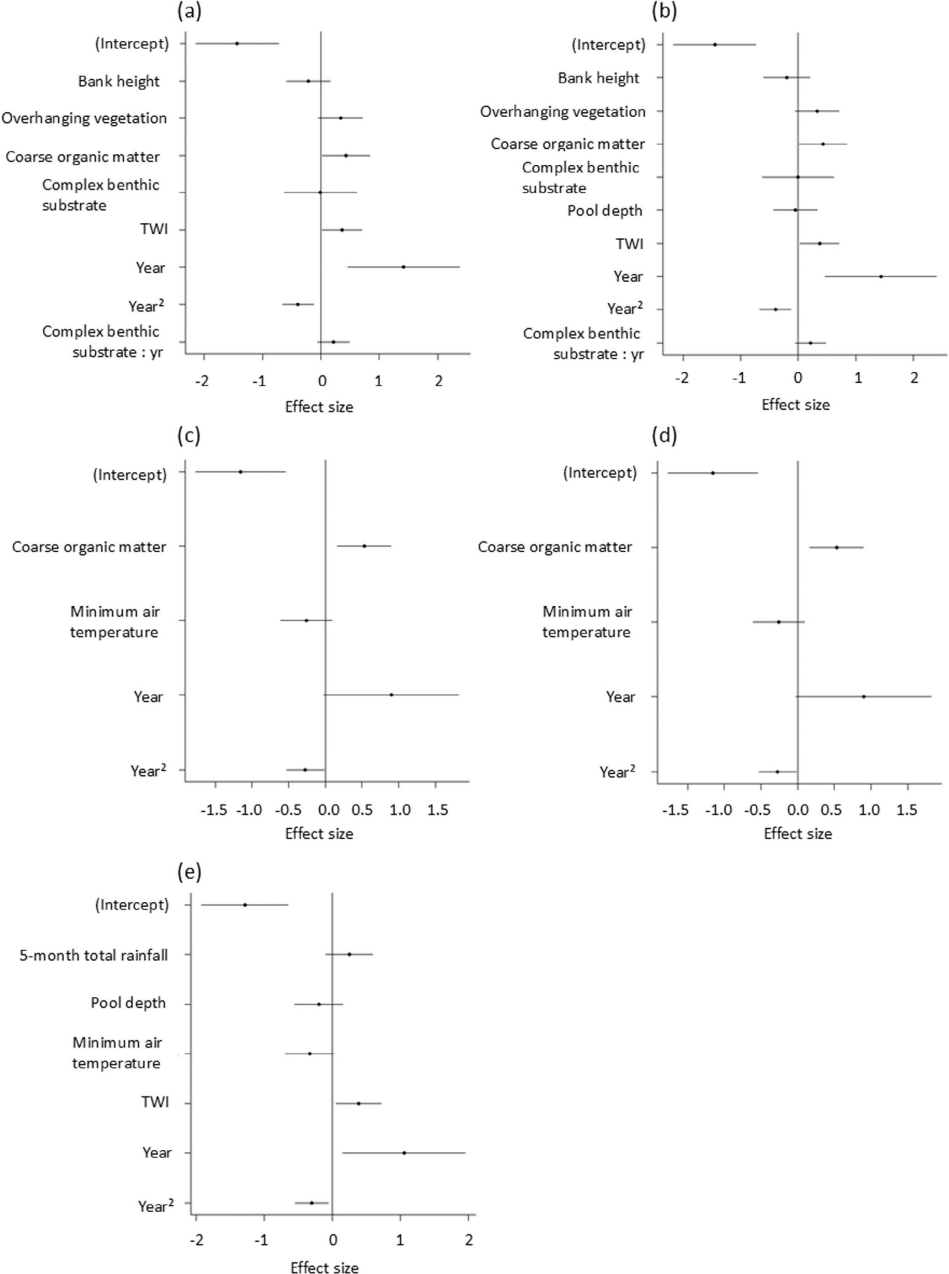
Effect sizes (coefficients) and 95% confidence intervals for variables in each of the five urban stream syndrome models: (a) channel morphology and stability, (b) flashier flows, (c) dominant tolerant species, (d) nutrients and contaminants and (e) water availability. The top‐ranked model (a) included variables representing channel morphology and stability (see Figure 3 for model estimates).

Of variables in the first‐ranked model, there were important effects of coarse organic matter (estimate = 0.427; confidence interval = 0.021–0.834), TWI (estimate = 0.365; confidence interval = 0.026–0.704) and year in its quadratic form (estimate = −0.394; confidence interval = −0.660 to −0.128) (Table S4). The probability of platypus occurrence was highest in Years 2017 and 2018 (Figure 3e). Confidence intervals for the other terms overlapped zero (Figure 4a; Table S4). The probability of platypus occurrence was not strongly influenced by bank height (Figure 3b) or overhanging vegetation (Figure 3c and Figure 4a). The effect size of the interaction between platypus occurrence complex benthic substrate and year was small (Figure 4a; Table S4). However, this term showed a trend for the positive effect of complex benthic substrate to get stronger over time (Figure 3f).

## 4 Discussion

Freshwater ecosystems are impacted by urbanisation and climate change, threatening species that depend upon them. This study used eDNA sampling to reveal habitat and environmental variables which influence platypus distribution in south‐east Queensland. We found positive effects of topographic wetness index, coarse organic matter and complex benthic substrate on platypus occurrence (the latter becoming stronger over time). There was also some temporal variability that appeared unrelated to rainfall patterns: Platypus probability of occurrence was greater in Years 2017 and 2018, indicating some unmeasured temporal influences (discussed below).

Topographic wetness index relates to slope and catchment area and gives an estimate of how likely a catchment area is to be wet (Gallant and Austin 2012). The index relates to the hydrological processes within a freshwater system, such as soil moisture which influences environmental processes and water availability (Daly and Porporato 2005). Topographic wetness index had a notable influence on platypus occurrence in this study: Sites with high relative wetness were more likely to have platypus. This signals the susceptibility of platypus to changing hydrological regimes in the study region. Changes in temperature and precipitation will alter catchment wetness, as evaporation will affect water availability and disrupt flow regimes (Bunn and Arthington 2002; Nickus et al. 2010; Jiménez Cisneros et al. 2014; Jia et al. 2019). A decrease in water and flow will reduce habitat availability and foraging area, increasing population fragmentation and potentially platypus genetic diversity (Grant and Temple‐Smith 2003; Klamt, Thompson, and Davis 2011). A warming, drier and shallower system will also impact platypus food sources, potentially forcing them over land where they would be at an increased risk of predation (Grant 2007; Klamt, Thompson, and Davis 2011). For platypus, water availability impacts movement and availability of foraging resources, as well as persistence of refuge areas. Therefore, water management and policies should prioritise the flow and amount of water to ensure platypus persisting.

Coarse organic matter was positively associated with platypus occurrence which relates to platypus prey as organic matter is habitat and food for aquatic macroinvertebrates (Serena et al. 2001). However, an increase in organic matter due to dominant invasive plant species can negatively affect the diversity of macroinvertebrates by changing water parameters due to the increase in leaf breakdown which contributes to increased nutrients (Walsh et al. 2005). Therefore, reducing the quality of habitat and the likelihood of platypuses occurring. The interaction between year and complex benthic substrate was not strong, but there was a tendency for the effect of complex benthic substrate to be most pronounced in 2020. In other regions, platypus favour sites with more complex benthic substrates as it increases the abundance and diversity of aquatic macroinvertebrates (Serena et al. 2001; Grant 2007, 2014). Four of the five positive platypus DNA records in 2020 scored < 50% in the habitat assessment for this variable. The urban stream syndrome can impact complex benthic substrate as bank erosion increases instream sediment, smothering the substrate and prey habitat (Grant and Temple‐Smith 2003). This disturbance of food resources could lead to local declines in platypus populations. Year in its quadratic form was related to platypus occurrence, most likely reflecting that the number of sites sampled increased as well as platypus detections, which subsequently decreased in Year 2020 (25 sites with 5 positive sites for platypus presences, Table S5). Further research is needed to explore the temporal factors influencing platypus occupancy.

We did not find any strong associations between platypus occupancy and other variables. This may be due to limited spatial variability across our study region, where sites have largely similar climatic conditions. Other variables not included in in this study might also have differed in local attributes, such as microclimate, landscape, hydrological processors, flow regimes, water quality and riparian vegetation composition (McIntosh et al. 2013; Millington, Lovell, and Lovell 2015; Booth et al. 2016). These variables should be considered in future research and management for platypus.

A more comprehensive survey, including a larger sample size and a broader range of waterways and land uses, would be recommended to more fully capture the variability in platypus habitat use. This approach would provide a more reliable basis for identifying key variables that influence platypus occurrence in the region. We used a rapid habitat assessment of a small section of the waterway, which might not fully represent how a platypus interacts with the entire system. The habitat assessment we used required some subjectivity in scoring which, although standardised, might still limit the relevance of the habitat survey. We also acknowledge that water eDNA does not necessarily pinpoint the exact location of a species' activity (Goldberg et al. 2016), but we addressed this by using multiple sampling points along each waterway. Despite these limitations, our research highlights potential priority variables which can be further investigated to inform the development of effective management practices for freshwater systems in the region.

This study makes an important contribution to our understanding of an iconic species that is threatened by increasing pressures on its freshwater habitat. As urbanisation continues to threaten freshwater ecosystems and biodiversity, it is important to understand how platypus distribution is affected by complex interactions between habitat and environmental variables. Conservation actions can be taken to protect or rehabilitate riparian zones, for example, by mitigating stormwater flow into freshwater systems to reduce compounding degrading impacts such as erosion and sedimentation. Our study has demonstrated how eDNA sampling can be incorporated with habitat and environmental assessments to understand which variables drive species persistence so that freshwater ecosystems can be restored. Future research should investigate other hydrological aspects that impact platypus such as flow regimes as this could affect platypus connectivity. The strong influence of topographic wetness that we found suggests that sustainable water is a priority. Innovations to sustain water in the environment are needed, such as nature‐based solutions of reusing treated greywater to replenish waterways when required (Boano et al. 2020). As Brisbane continues to grow and put stress on freshwater waterways, future research should focus on platypus population viability in relation to habitat size and the multiple stressors that impact the future of platypus in the region. With their wide spatial distribution, it is important for platypus to remain a priority species for monitoring and research. Although the species has proven robust to existing stressors, its cryptic nature makes it a difficult species to monitor for population declines (Hawke, Bino, and Kingsford 2020). It is vital that a species such as the platypus, which is evolutionarily distinct and iconic, is conserved.

## Author Contributions

**Tamielle Brunt:** data curation (equal), formal analysis (equal), investigation (equal), methodology (equal), project administration (equal), writing – original draft (equal), writing – review and editing (equal). **Matt Cecil:** funding acquisition (equal), methodology (equal), project administration (equal). **Josh Griffiths:** methodology (equal), project administration (equal), writing – review and editing (supporting). **Christine Adams‐Hosking:** supervision (supporting), writing – review and editing (supporting). **Peter J. Murray:** conceptualization (supporting), supervision (supporting), writing – review and editing (supporting). **Annabel L. Smith:** data curation (equal), formal analysis (equal), investigation (equal), supervision (lead), writing – review and editing (equal).

## Conflicts of Interest

J.G. is an employee of EnviroDNA that analysed eDNA samples. The other authors declare that they have no known competing financial interests or personal relationships that could have appeared to influence the work reported in this paper. Open access publishing facilitated by The University of Queensland, as part of the Wiley ‐ The University of Queensland agreement via the Council of Australian University Librarians.

## Supporting information


Appendix S1

## Data Availability

The datasets generated and/or analysed during the current study are available on the Zenodo Public Repository https://zenodo.org/doi/10.5281/zenodo.10595290.
