# Supplementary material for: Environmental DNA Reveals Habitat Variables Driving Platypus (Ornithorhynchus anatinus) Distribution Across an Urbanised Landscape
Source: Ecol Evol. 2025 Jan 9;15(1):e70783. doi: 10.1002/ece3.70783 (PMC11717483; doi:10.1002/ece3.70783)
Supplement: Supplementary file 1 — Appendix S1 [file ECE3-15-e70783-s001.docx]

Supplementary information to “Environmental DNA reveals habitat variables driving platypus (*Ornithorhynchus* *anatinus*) distribution across an urbanised landscape.” Submitted to *Ecology and Evolution* by: Tamielle Brunt, Matt Cecil, Josh Griffiths, Christine Adams-Hosking, Peter J. Murray and Annabel L. Smith.

Corresponding author: Tamielle Brunt, School of Agriculture and Food Sustainability and the School of Environment, The University of Queensland, Gatton College QLD 4343, Australia. Email: [tamielle.brunt@uq.net.au](mailto:tamielle.brunt@uq.net.au)

**Table S1**. Number of sites sampled between 1 and 5 times (dataset on Zenodo https://zenodo.org/doi/10.5281/zenodo.10595290.

| Number of times sampled per site | Number of sites | Proportion % |
| --- | --- | --- |
| 1 | 114 | 62 |
| 2 | 33 | 18 |
| 3 | 19 | 10 |
| 4 | 15 | 8 |
| 5 | 4 | 2 |
| Total | 185 | 100 |

**Table S2.** Habitat and environmental variables used to model platypus distribution. Habitat variables were collected in the field and environmental variables were collected from remotely sensed databases.

| **Variable** | **Influence on platypus** | **References** | **Chosen for final analysis (yes / no) with**  **Pearson correlation coefficient (r)** |
| --- | --- | --- | --- |
| **Habitat** | | | |
| Vegetation | | | |
| - Native large-medium sized trees on banks | - Consolidation of banks with root system - Burrow entrances hidden among roots - Organic input into aquatic ecosystem | Bryant (1993); Woon (1995); Rohweder & Baverstock (1999); Serena et al. (1998, 2001); Grant (2004, 2014) | No, correlated with:   - Overhanging vegetation r = 0.40 - Large woody debris r = 0.40 - Bank height r = 0.36 - Earthen banks r = 0.36 - Coarse organic matter r = 0.30 |
| - Overhanging | - Shade and shelter - Conceal burrow entrances - Organic input into aquatic ecosystem | Bryant (1993); Woon (1995); Serena et al. (1998); Grant (2014) | Yes |
| - In-stream large woody debris | - Habitat for macroinvertebrates | Bryant (1993); Serena et al. (2001); Grant (2014) | No, correlated with:   - Concave banks r = 0.36 - Bank height r = 0.35 |
| - Coarse organic matter | - Favourable habitat for benthic invertebrate prey | Grant (2014) | Yes |
| Water |  |  |  |
| - Water depth | - Between 1 and 5 m are effective foraging depths | Serena et al. (1998); Grant (2007, 2014); Brunt et al. (2018) | Yes |
| Bank |  |  |  |
| - Bank height | - Minimum of 0.5 m necessary for burrow construction | Bryant (1993); Woon (1995); Grant (2007, 2014) | Yes |
| - Earthen banks | - Burrow construction and maintenance | Grant (2014) | No, correlated with:   - Bank height r = 0.64 - Concave banks r = 0.65 - Large to medium sized trees r = 0.36 |
| - Absence of erosion | - Burrow maintenance - Reduced in-stream sedimentation | Grant (2014) | No, correlated with:   - Overhanging vegetation r = 0.32 |
| - Consolidated banks | - Maintenance of burrows - Reduced in-stream sedimentation | Serena (1994); Grant (2014) | No, correlated with:   - Bank height r = 0.49 - Earthen banks r = 0.61 - Concave banks r = 0.49 - Large–medium sized trees r = 0.36 |
| - Concave or near vertical banks | - Secure access to burrow - Hide entrance - Lower predation risk | Serena et al. (1998) | No, correlated with:   - Bank height r = 0.78 - Earthen banks r = 0.65 |
| Substrate | Complexity increases food abundance | Grant (2004; 2014) |  |
| - Cobbled (complex benthic substrate) | - Favourable habitat for benthic invertebrate prey | Serena et al. (2001); Grant (2007; 2014) | Yes |
| - Fine soils substrate |  | Milione & Harding (2009) |  |
| **Environment** |  |  |  |
| Temperature   - Air (minimum and maximum) | - Platypus cannot withstand environmental temperatures >30°C - Avoid extreme heat - Thermal suitability - annual maximum temperature physiological impacts | Robinson (1954)  Grant & Dawson (1978); Bethge et al. (2004)  Klamt et al. (2011) | Yes, minimum |
| Elevation | - Influences catchment water - Maximum river segment elevation (21% positive association) | Bino et al. (2020) | No |
| Precipitation   - Total annual rainfall - Antecedent rainfall (rainfall total in the five months prior to breeding season) | - Platypus highly water-dependent | Serena et al. (2014) | Yes, antecedent rainfall |
| Slope  - Topographic Wetness Index | - >20° necessary for burrow construction - Chosen to be an environmental variable that may influence platypus because it estimates relative wetness within a catchment which would relate to water availability within the areas. It is calculated as log_e (specific catchment area/slope). | Woon (1995); Grant (2004) | Not specifically – within the TWI  Yes, slope included. |

**Table S3**. Habitat Quality Index (based on Grant 2014) used to assess platypus habitat quality at each of the 185 eDNA sample sites. A score between 0 and 4 was given for each variable: 0 = none, 1 = <25%, 2 = 25–49%, 3 = 50–74%, 4 = >75%).

| **Habitat variable** | **Known or potential benefit to platypus** | **SCORE** |
| --- | --- | --- |
| **Bank variables** | | |
| Consolidated banks | Maintenance of burrows, reduced in-stream sedimentation |  |
| Large-medium sized trees on banks | Consolidation of banks, organic input to aquatic ecosystem |  |
| Overhanging vegetation <2 m above water | Consolidation of banks, organic input to aquatic ecosystem, lower predation risk due to shelter while foraging and entering/leaving burrows |  |
| Earthen banks | Allows construction and maintenance of burrows |  |
| Bank height >1 m | Preferred bank morphology for construction of burrows and maintenance |  |
| Concave or near vertical banks | Secure access to burrow, hide entrance, lower predation risk |  |
| Absence of erosion | Maintenance of burrows, maintenance of riparian vegetation, reduced in-stream sedimentation |  |
| **In-stream variables** | | |
| Pool depth (>1 m but<5 m) | Preferred foraging depth for platypus, lower risk of predation |  |
| Large woody debris (LWD, >10 cm diameter) | Habitat and food for benthic invertebrate prey |  |
| Complex benthic substrate (cobbled, gravel) | Favourable habitat for benthic invertebrate prey |  |
| Coarse organic matter – if visible | Favourable habitat for benthic invertebrate prey |  |

**Table S4.** Coefficients (Estimate), standard error, z value, P value and 95% confidence intervals for variables included in the top-ranked model related to channel morphology, of platypus occurrence in south-east Queensland, Australia.

|  | | | | | | 95% Confidence intervals | |
| --- | --- | --- | --- | --- | --- | --- | --- |
|  | Estimate | Std. Error | z value | P value | Lower | | Upper |
| (Intercept) | -1.433 | 0.359 | -3.991 | < 0.001 | -2.137 | | -0.729 |
| Bank height | -0.214 | 0.193 | -1.113 | 0.266 | -0.592 | | 0.163 |
| Overhanging vegetation | 0.335 | 0.193 | 1.733 | 0.083 | -0.044 | | 0.713 |
| Coarse organic matter | 0.427 | 0.208 | 2.059 | 0.039 | 0.021 | | 0.834 |
| Complex benthic substrate | -0.008 | 0.313 | -0.026 | 0.979 | -0.622 | | 0.606 |
| Topographic Wetness Index | 0.365 | 0.173 | 2.110 | 0.035 | 0.026 | | 0.704 |
| Year | 1.419 | 0.490 | 2.895 | 0.004 | 0.458 | | 2.379 |
| Year² | -0.394 | 0.136 | -2.899 | 0.004 | -0.660 | | -0.128 |
| Complex benthic substrate: year | 0.215 | 0.136 | 1.577 | 0.115 | -0.052 | | 0.482 |

Table S5: Annual platypus eDNA detection efforts, including the number of sampling sites, the number of positive detections, and the proportion of detections as a percentage of sites sampled.

| Year | Number of sites sampled | Number of positive platypus DNA detections | Proportion of detections % |
| --- | --- | --- | --- |
| 2016 | 59 | 12 | 20.3 |
| 2017 | 25 | 12 | 48.0 |
| 2018 | 22 | 13 | 59.1 |
| 2019 | 54 | 12 | 22.2 |
| 2020 | 25 | 5 | 20.0 |
| Total | 185 | 54 | 29.2 |

**
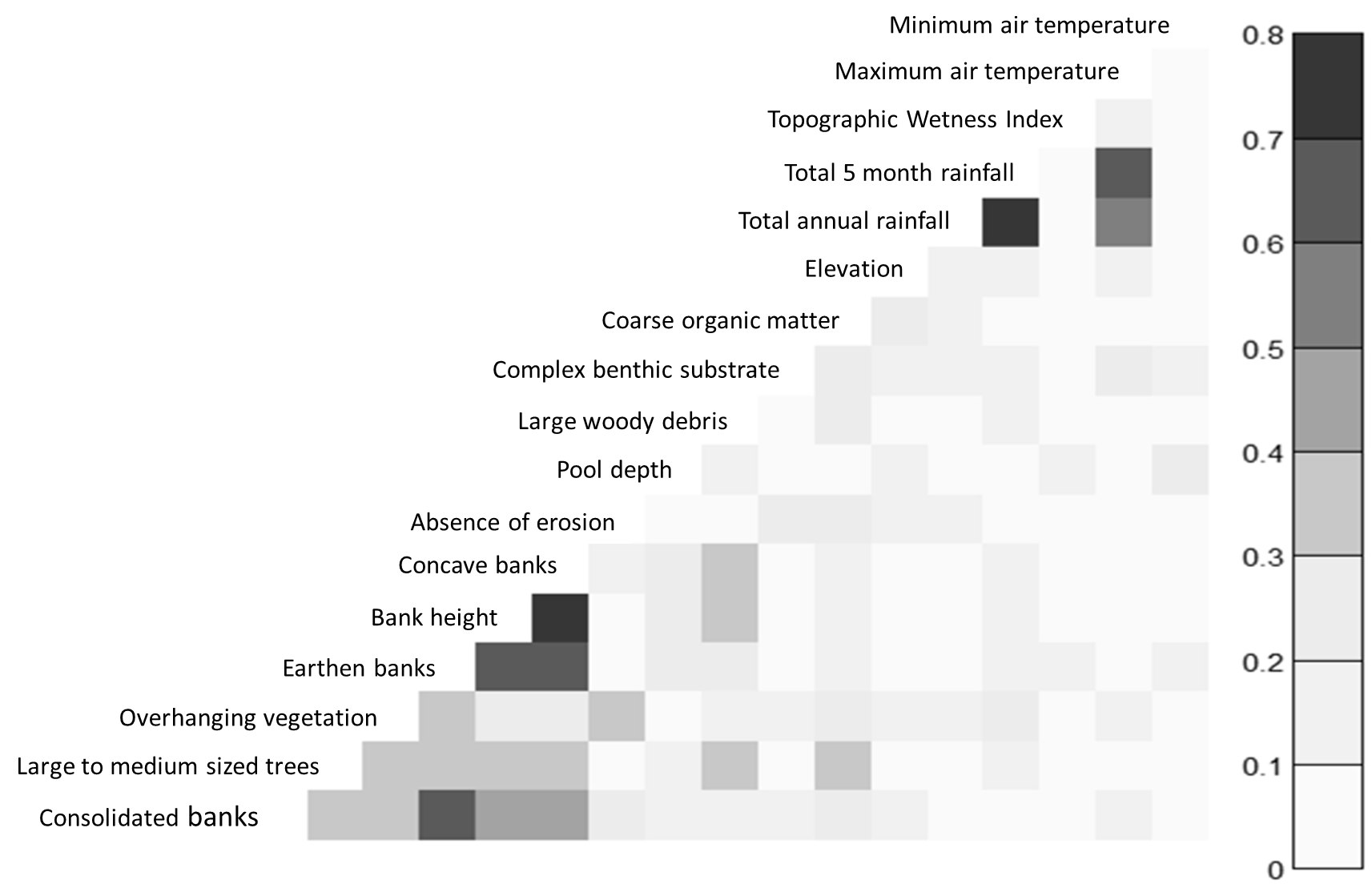
**
**Figure S1**. Correlation coefficients (*r*) among 17 habitat and environmental variables, giving 136 coefficients indicated by the shading. When a pair of variables was correlated (*r* > 0. 30) we excluded one of the variables from subsequent analysis, retaining the variable with the greatest potential to influence platypus distribution. Absolute correlation coefficients are shown and do not indicate the direction of the effect.

|  | 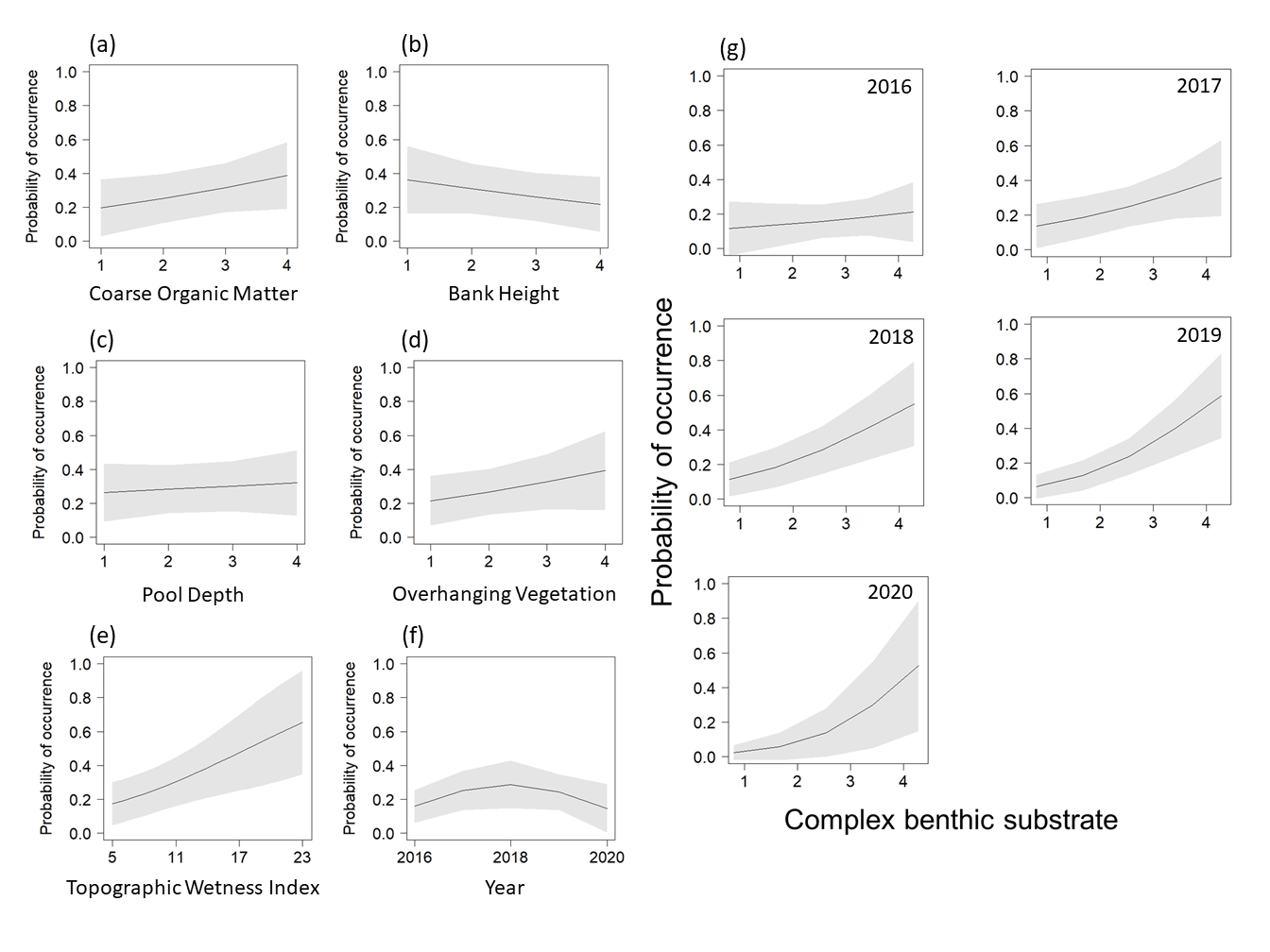 |
| --- | --- |

**Figure S2.** Model estimates and 95% confidence intervals for the second-ranked model of platypus occurrence, including variables related to flashier flows. The model included effects of (a) coarse organic matter, (b) bank height, (c) pool depth, (d) overhanging vegetation, (e) topographic wetness index, (f) year, and (g) interaction between year and complex benthic substrate.

**References**

Bethge, P., Munks, S., Otley, H. and Nicol, S. (2004). ‘Platypus burrow temperature at a Subalpine Tasmanian Lake’, *Proceedings of the Linnean Society of New South Wales*,125, 273 – 276.

Bino, G., Kingsford, R.T. & Wintle, B.A. (2020). 'A stitch in time – Synergistic impacts to platypus metapopulation extinction risk', *Biological* *Conservation*, 242, 108399.

Brunt, T., Adams-Hosking, C. & Murray, P. (2018). ‘Prime real estate for the platypus (*Ornithorhynchus* *anatinus*): habitat requirements in a peri-urban environment’, Queensland Naturalist, 56, 4 – 6.

Bryant, A.G. (1993). ‘*An evaluation of the habitat characteristics of pools used by platypuses, (Ornithorhynchus anatinus) in the upper Macquarie River system, N. S.W*.’, Honour’s thesis, Charles Sturt University, Mitchell Bathurst.

Grant, T.R. (2004). ‘Depth and Substrate Selection by Platypuses, *Ornithorhynchus* *anatinus*, in the Lower Hastings River, New South Wales’, *Proceedings of the Linnean Society of New South Wales*, 125, 235 – 241.

Grant, T. (2007). Platypus, 4th edn, CSIRO Publishing, Collingwood.

Grant, T. (2014). The platypus and the environmental impact assessment process: more cogitations of a consultant. Consulting Ecology 33, 50 - 63.

Grant, T.R., & Dawson, T.J. (1978). ‘Temperature regulation in the platypus, *Ornithorhynchus* *anatinus*, Maintenance of body temperature in air and water’, *Physiological Zoology*, 51, 1 – 6.

Grant, T.R. & Temple-Smith, P.D. (2003). ‘Conservation of the platypus, *Ornithorhynchus* *anatinus*: Threats and challenges’, *Aquatic Ecosystem Health and Management*, 6, 5 – 18.

Klamt, M., Thompson, R., & Davis, J. (2011). ‘Early response of the platypus to climate warming’, *Global Change Biology*, 17, 3011 - 3018.

Milione, M., & Harding, E. (2009). ‘Habitat use by platypus (*Ornithorhynchus* *anatinus*) in a modified Australian Wet Tropics catchment, north-eastern Queensland’, *Australian Mammalogy*, 31, 35 – 46.

Robinson, K.W. (1954). ‘Heat tolerances of Australian monotremes and marsupials’, *Australian Journal of Biological Science*, 7, 348 – 260.

Rohweder, D.A., & Baverstock, P.R. (1999). ‘Distribution of platypus *Ornithorhynchus* *anatinus* in the Richmond River Catchment, northern New South Wales’, *Australian Zoologist*, 31, 30 – 37

Serena, M. (1994). ‘Use of time and space by platypus (*Ornithorhynchus* *anatinus*: Monotremata) along a Victorian stream’, *Journal of Zoology*, 232, 117 – 131.

Serena, M., Thomas, J.L., Williams., G.A. & Officer, R.C.E. (1998). ‘Use of stream and river habitats by platypus, *Ornithorhynchus* *anatinus*, un an urban fringe environment’, *Australian Journal of Zoology*, 46, 267 – 282.

Serena, M., Worley, M., Swinnerton, M. & Williams G.A. (2001). ‘Effect of food availability and habitat on the distribution of platypus (*Ornithorhynchus* *anatinus*) foraging activity*’, Australian Journal of Zoology*, 49, 263 – 277.

Serena, M., Williams, G.A., Weeks, A.R. & Griffiths, J. (2014). ‘Variation in the platypus (*Ornithorhynchus* *anatinus*), life-history attributes and population trajectories in urban streams’, *Australian Journal of Zoology,* 62, 223 – 234.

Woon, P.M.N. (1995). ‘*Habitat preferences of platypus (Ornithorhynchus anatinus Shaw 1799) in the Hastings River catchment, N.S.W’,* Unpublished Master thesis, University of Sydney, Sydney.
